# Effects of Supplement of *Marichromatium gracile* YL28 on Water Quality and Microbial Structures in Shrimp Mariculture Ecosystems

**DOI:** 10.3390/genes12010040

**Published:** 2020-12-30

**Authors:** Liang Cui, Bitong Zhu, Xiaobo Zhang, Zhuhua Chan, Chungui Zhao, Runying Zeng, Suping Yang, Shicheng Chen

**Affiliations:** 1 Department of Bioengineering and Biotechnology, Huaqiao University, Xiamen 361021, China; liangcuiv5@163.com (L.C.); BitongZhu@yahoo.com (B.Z.); caitcadam@163.com (X.Z.); chungui@hqu.edu.cn (C.Z.); 2 State Key Laboratory Breeding Base of Marine Genetic Resource, Third Institute of Oceanography, Ministry of Natural Resources, No. 178 Daxue Road, Xiamen 361005, China; chan@tio.org.cn (Z.C.); zeng@tio.org.cn (R.Z.); 3 Department of Biomedical Diagnostic and Therapeutic Sciences, School of Health Sciences, Oakland University, Rochester, MI 48309, USA

**Keywords:** *Marichromatium gracile* YL28, microbial community, bioremediation, probiotics, shrimp mariculture ecosystem

## Abstract

The elevated NH_3_-N and NO_2_-N pollution problems in mariculture have raised concerns because they pose threats to animal health and coastal and offshore environments. Supplement of *Marichromatium gracile* YL28 (YL28) into polluted shrimp rearing water and sediment significantly decreased ammonia and nitrite concentrations, showing that YL28 functioned as a novel safe marine probiotic in the shrimp culture industry. The diversity of aquatic bacteria in the shrimp mariculture ecosystems was studied by sequencing the V4 region of 16S rRNA genes, with respect to additions of YL28 at the low and high concentrations. It was revealed by 16S rRNA sequencing analysis that Proteobacteria, Planctomycete and Bacteroidetes dominated the community (>80% of operational taxonomic units (OTUs)). Up to 41.6% of the predominant bacterial members were placed in the classes Gammaproteobacteria (14%), Deltaproteobacteria (14%), Planctomycetacia (8%) and Alphaproteobacteria (5.6%) while 40% of OTUs belonged to unclassified ones or others, indicating that the considerable bacterial populations were novel in our shrimp mariculture. Bacterial communities were similar between YL28 supplements and control groups (without addition of YL28) revealed by the β-diversity using PCoA, demonstrating that the additions of YL28 did not disturb the microbiota in shrimp mariculture ecosystems. Instead, the addition of YL28 increased the relative abundance of ammonia-oxidizing and denitrifying bacteria. The quantitative PCR analysis further showed that key genes including *nifH* and *amoA* involved in nitrification and nitrate or nitrite reduction significantly increased with YL28 supplementation (*p <* 0.05). The supplement of YL28 decreased the relative abundance of potential pathogen *Vibrio*. Together, our studies showed that supplement of YL28 improved the water quality by increasing the relative abundance of ammonia-oxidizing and denitrifying bacteria while the microbial community structure persisted in shrimp mariculture ecosystems.

## 1. Introduction

The intensive culture and high additive inputs (such as antibiotics and other chemicals) in aquaculture systems have increased coastal and offshore pollution, which poses a serious threat to the environment and human health [1]. Studies have shown that the sediment–water interface (SWI) is the primary location where microorganisms convert fish excreta and feed residues (organic nitrogen compounds) into inorganic nitrogen such as ammonia and nitrite [2]. Microbial modulators (such as probiotics or potential probiotics) have been used to alleviate these problems [3]. Major categories in commercial formulations of aquaculture probiotics include *Bacillus* spp., lactic acid bacteria (LAB), anoxygenic phototrophic bacteria (APB), *Bdellovibrio*, *Nitrobacter*, *Pseudomonas* and many others [3]. Numerous studies have revealed that supplementation of *Bacillus* to the diet improved growth performance, digestive enzyme activities, antioxidant function, immune responses, and disease resistance of fish [4]. *Rhodopseudomonas palustris* G06 improved the growth performance of juvenile white shrimp when administered as a water additive [5]. *Pseudoalteromonas sp.* BC228 improved the digestive enzyme activities, immune response and disease resistance of juvenile sea cucumber [6]. Among them, APB have been one of the most common probiotic agents in China since the 1980s. Moreover, purple non-sulfur bacteria (PNSB), a group of APB, have been also widely applied. For example, supplements of *Rhodopseudomonas palustris*, *Rubrivivax gelatinosa*, *Rhodobacter capsulata*, or *Rhodobacter sphaeroides* improved water quality, reduced the morbidity and mortality rates, and stimulated the growth of the aquatic animals [7,8]. Despite their extensive applications in aquaculture, most APB are limited in freshwater rearing industry (due to their salinity tolerance). Some strains such as *Marichromatium purpuratum* RuA2 [9] and *M. gracile* YL28 [10] could thrive in a salt environment. The salt-tolerant genes and fates of these probiotics in the aquaculture ecosystems remain to be further investigated. 

Our previous studies showed that *M. gracile* YL28 (isolated from mangrove special environment) utilized ammonia, nitrite (up to 200 mg/L) or nitrate as the sole nitrogen source for cell growth and efficiently eliminated these chemicals [11,12,13]. Y28 can grow at wide ranges of salt (0.5–5.5% NaCl), pH (5.7–8.5) and temperature (20–35 °C). It grows photoautotrophically and anaerobically in the presence of sulphide, sulphur, thiosulfate or sulfite as electron donor. Moreover, it can also grow photoheterotrophically with various organic substrates [10]. YL28 performs simultaneous heterotrophic nitrification and denitrification under anaerobic conditions [14]. Further studies reveal that YL28 has six nitrogen cycle and three sulfur cycle pathways, which allows YL28 to remove high concentrations of nitrite by the combination of denitrification, complete assimilation nitrate reduction and fermentative nitrate reduction (DNRA) as well as toxic sulfur compounds (i.e., H_2_S). Some unique stress response genes facilitate YL28 to tolerate the high salinity environment [15,16]. The accumulative survival rates of *Oryzias melastigma* were 100% with YL28 supplement. This strain is non-toxic to experimental animals by the acute toxicity tests and the gross anatomy observation. In addition, YL28 had significant growth-promoting effects, which increased the weight and length in shrimps [13]. It is promising to use YL28 as a model to represent APB for studying their effects as probiotics. Thus, it is worth investigating whether YL28 can function as a probiotic and how its application affects the indigenous microbial community structures in marine aquaculture ecosystem [17].

The objectives of the present study were as follows: (1) to examine the impact of YL28 supplements on water quality; (2) to evaluate if YL28 can be a safe probiotic modulating microbial community structure in marine aquaculture system (especially for beneficial or pathogenic microorganisms); (3) to investigate the interactions between YL28 and other nitrogen transformation organisms.

## 2. Materials and Methods

### 2.1. Preparation of Strains and Suspensions

*Marichromatium gracile* YL28 was chosen for this study due to its efficient transformation of nitrogen and sulfur compounds [10]. Pfennig medium was used with some modifications (1 L): sodium acetate (24.3 mmol/L), seaweed oligosaccharides (0.2%, *w*/*v*) and yeast extract (0.1%, *w*/*v*) were added to the basic medium; 1.5 mmol/L of Na_2_S·9H_2_O was substituted by 0.75 mmol/L of Na_2_S_2_O_3_ [14]. YL28 was anaerobically cultured under 3000 lx, at 28 °C for 4 days. Cells were washed with 2.0% sterile NaCl solution for three times by centrifuging at 4500 *g* for 8 min. The final concentration of bacterial cells was adjusted to 9.4 × 10^9^ CFUs/mL.

### 2.2. The Sample Treatment, Experimental Design and Management

Water and sediment samples were collected from five separate sites (with the minimum distance ranging from 20 to 30 m) of the shrimp rearing ponds (23°38’34.5’ N, 117°23’48” E, Xingchen town, Zhangzhou City, Fujian Province, China). These samples (referred to CK-0) were immediately placed on dry ice before transporting to our laboratory. We reconstituted the experimental ecosystems by first aliquoting the sediments into 9 containers (25.0 cm length × 25.0 cm width × 25.0 cm height). To each container, 14.0 L of seawater from the rearing ponds were added. The collected sediments were reconstituted with a height of 3.0 cm in our experimental systems. The bacterial cells of 3.0 × 10^6^ cells/mL or 1.5 × 10^8^ cells/mL were added to above containers as the low (YL28-L) or high-dose (YL28-H) treatment, respectively. The control group (CK) was the same as the above treatments without YL28 cells. Each treatment had at least triplicates. YL28 cells were supplemented to the above systems every 6 days to maintain enough cell concentrations. These treatments were maintained under 28 °C and natural light in room.

### 2.3. Physicochemical Parameters Analyses

Sediments were briefly washed with 2.0 mol/L of KCl solution (Sediment: KCl = 1:5, WV) by centrifuging at 4500 *g* for 10 min. After centrifuging, the supernatant (100 mL) was filtrated through a sterile filter (0.22 μm). For chemistry parameter measurements, we used the rearing water samples (20 mL). Ammonia, nitrate and nitrite concentrations were determined following the procedures described previously [14]. Total nitrogen (TN), dissolved total phosphorus (DTP) and chemical oxygen demand (COD) were measured by using potassium persulphate oxidation-UV spectrophotometry (GB11914-89), molybdenum antimony anti-spectrophotometry (GBT11893-89) and permanganate index (GB11914-89), respectively. The removal rate of inorganic nitrogen was calculated as follows: *r* % = (C_0_ − C_t_)/C_0_ × 100%, where *r* is the residual rate of inorganic nitrogen; C_t_ and C_0_ are the concentrations of inorganic nitrogen at the time of measurement and at the start of the experiment. β-diversity was estimated by using PCA, PCoA and nMDS analysis (based on Permanova test) at the genera level. The interactions among the environmental factors (e.g., ammonia, nitrite, nitrate, TN, DTP and COD) and bacterial community (genus level) were analyzed using canonical correspondence analysis (CCA). The statistical analysis was conducted by using one-way analysis of variance (ANOVA) followed by a multiple comparison (LSD-test). Significant difference was set at *p <* 0.05.

### 2.4. DNA Extraction and Sequencing

Microbial DNA was extracted from sediment samples using the HiPure Soil DNA D3142 Kits (Magen, Guangzhou, China) according to manufacturer’s protocols. Amplicon tagging and sequencing were conducted at Guangzhou Gene Denovo Biotechnology Co., Ltd. (China). The 16S rDNA V3-V4 hypervariable region was amplified with the microbial genomic DNA using primers 341F (5′- CCTACGGGNGGCWGCAG -3′) and 806R (5′- GGACTACHVGGGTATCTAAT -3′). The PCR mixture contained 5 μL of 10 × KOD (DNA polymerase from *Thermococcus kodakaraensis*) buffer, 5 μL of 2.5 mmol/L dNTPs, 1.5 μL of each primer (5 μmol/L), 1 μL of KOD DNA polymerase, and 100 ng of microbial DNA. The PCR conditions were set as the following: 95 °C for 2 min, followed by 27 cycles at 98 °C for 10 s, 62 °C for 30 s, and 68 °C for 30 s and a final extension at 68 °C for 10 min. Amplicons were extracted from 2% agarose gels and purified using the AxyPrep DNA Gel Extraction Kit (Axygen Biosciences, Union City, CA, USA) according to the manufacturer’s instructions. The concentration of DNA was quantified using ABI StepOnePlus Real-Time PCR System (Life Technologies, Foster City, CA, USA). Purified amplicons were pooled in equimolar and paired-end sequenced (2 × 250 bp) on an Illumina platform according to the standard protocols.

### 2.5. qPCR Analysis

Primers used for amplification of bacterial 16S rRNA genes (ammonia-oxidizing archaea (AOA) and ammonia-oxidizing bacteria (AOB), nitrogen-fixing gene (*nif*H), nitrification gene (*amo*A), denitrification genes (*nos*Z and *nar*G) are listed in Supplementary Table S1. Quantitative PCR amplification was performed in 50 μL reaction system which contained 1.0 μL of primers (10 μmol/L), 1.0 μL of template, 25.0 μL of 2 × Taq MasterMix (Cw0716, ComWin Biotech Co., Ltd., Beijing, China) and 23 μL of water. The quantitative PCR amplification was conducted as follows: pre-denaturation at 94 °C for 5 min, followed by 30 cycles of denaturation at 94 °C for 30 s, annealing at 55 °C for 30 s, and elongation at 72 °C for 30 s; and final extension at 72 °C for 10 min. The PCR products were cloned into T-vector and sequenced for confirmation.

### 2.6. DNA Sequence Analysis

Sequencing reads were analyzed using QIIME (v1.9.1) with the default parameters. Sequences with N proportion of more than 10% and base error rate greater than 1% were removed. FLASH (v1.2.11) was used to assemble the paired-end sequences [18]. Reads with homopolymers more than 8 bp, primer mismatches, ambiguous bases and sequence lengths less than 150 bp were further eliminated. The UCHIME in mothur software (v1.31.2) was used to remove the chimera sequences [19]. Uparse (usearch v9.2.64) software was used to cluster sequences in order to study the species composition diversity. Operational taxonomic unit (OTU) were counted for all samples with a cutoff of 97% identity. Rare OTUs (less than 1) were not used in the subsequent analysis. The representative sequences of each clustered OTU were selected according to the maximum length, aligned to Greengenes 16S rRNA gene database (v13.8) [20] and classified by RDP classifier (v.2.2) [21]. The α-diversity indices (Shannon and Chao1) were calculated for each sample. In order to exclude data analysis differences caused by sequence differences, the sequences of all samples were normalized to 11,476 sequences (Min: 11,476, Max: 12,300, Mean: 11,986, SD: 445). The relative abundance of each sample at each classification level was calculated and visualized by a histogram. Principal components analysis (PCA), principal coordinate analysis (PCoA) and non-metric multi-dimensional scaling analysis (nMDS) were conducted for β-diversity analysis.

### 2.7. Sequence Data Availability

Raw paired-end reads were submitted to the NCBI BioProject (accession number: PRJNA505047), to the NCBI BioSample (SAMN10408845) and to the NCBI Sequence Read Archive (SRS4036541).

## 3. Results

### 3.1. The Nitrogen Removal in Aquatic Ecosystems by YL28

**Figure 1:**
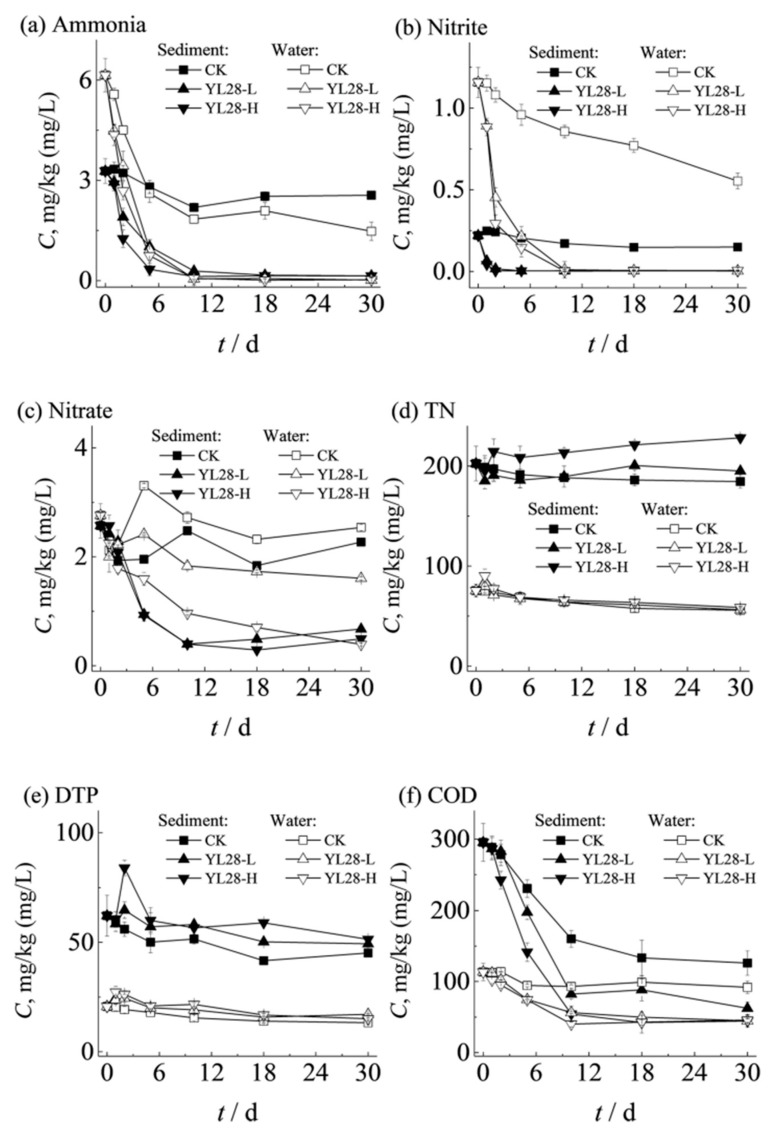
Effects of supplements of YL28 on the environmental factors of sediment and water column, respectively. The addition time of YL28 is on 0, 6th, 12th, 18th days, respectively. The data on 0 d is the determination value of samples without YL28 addition. CK, control group; YL28-L and YL28-H represent a low and high dose addition of YL28 into water with a concentration of 3.0 × 10^6^ cells/mL and 1.5 × 10^8^ cells/mL, respectively. (**a**) Comparison of ammonia concentration affected by YL28 additions. (**b**) Comparison of nitrite concentration affected by YL28 additions. (**c**) Comparison of nitrate concentration affected by YL28 additions. (**d**) Comparison of TN concentration affected by YL28 additions. (**e**) Comparison of total phosphate (DTP) concentration affected by YL28 additions. (**f**) Comparison of chemical oxygen demand (COD) affected by YL28 additions. Significant difference: *p* < 0.05.

In the CK group, the ammonia removal rates were 14.51% in the sediments and 57.60% in the water column after 5-day incubation for each. However, in the YL28-L group, the ammonia removal rates (Figure 1a) were 68.99% in the sediments and 85.00% in the water column. Addition of a higher concentration of YL28 cells (YL28-H) led to better ammonia removal rates (89.83 and 87.95% in the sediment and water column, respectively) than those in the YL28-L group under the same conditions. Remarkably, only trace amounts of ammonia were detected in the YL28-L and YL28-H groups (4.24–4.28% in sediment, 0.25–0.38% in water column, respectively). However, at least 77.87% of ammonia in the sediment and 23.95% in the water were observed in the CK group. The concentrations of nitrite in water also decreased with the incubation time (Figure 1b) though its concentrations remained unchanged in the sediments. After 10-day incubation, nitrite concentrations in YL28-L and YL28-H groups decreased to the undetectable level in both sediments and water columns, demonstrating that YL28 possessed the strong ability to metabolize nitrite. Nitrate concentrations (Figure 1c) in the sediments and water column remained relatively stable during testing periods (within 30 days) in the control group (CK). YL28-L only slightly contributed to the nitrate removal in the water column while YL28-H significantly decreased the nitrate concentration. However, addition of YL28 increased the concentrations of total nitrogen (TN, Figure 1d) and dissolved total phosphate (DTP, Figure 1e) in the sediments while not much was observed in the water. The COD removal trend was similar to that of ammonia (Figure 1f).

### 3.2. Effects of YL28 on Bacterial α- and β-Diversity

**Figure 2:**
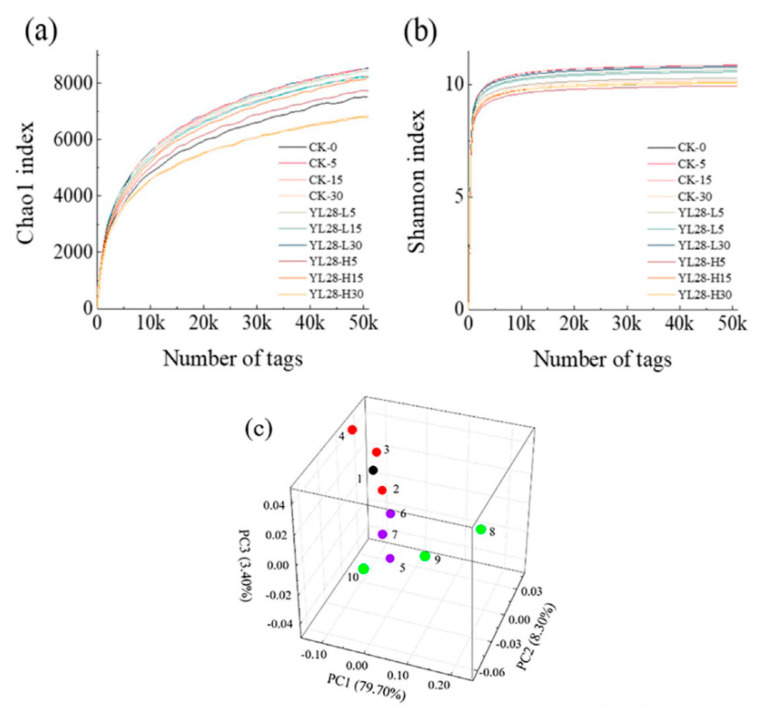
The indices of Chao1 and Shannon of bacterial community in sediment samples. (**a**) Number of reads to Chao1 index curve; (**b**) number of reads to Shannon index curve. Significant difference: *p <* 0.05; (**c**) Principal components analysis (PCA) of microbial communities. Colors and numbers were used to distinguish different systems and samples. Black: 1, CK-0. red: 2, CK-5; 3, CK-15; 4, CK-30. blue: 5, YL28-L5; 6, YL28-L15; 7, YL28-L30. Green: 8, YL28-H5; 9, YL28-H15; 10, YL28-H30. Significant difference: *p <* 0.05.

In sediment samples, α-diversity was estimated by richness indices (Chao1) and diversity indices (Shannon) (Figure 2a,b, Table 1 and Table 2). In control samples (CK-0, 5, 15, 30 d), both microbial richness (revealed by Chao1) and diversity (Shannon) indexes had no significantly change (*p >* 0.05). Compared to the control group (CK), no significant difference for microbial richness (Chao1) or diversity (Shannon) in YL28-L or YL28-H samples was also detected (*p >* 0.05).

PCA analysis showed that PC1, PC2 and PC3 explained 79.70%, 8.30% and 3.40% of the total variance, respectively; the cumulative variance accounted for 91.40%. On PC1 level of CK and YL28-L, no significant difference was found among samples with time. There was a negative association between them (PC1 level). On the PC2 level, CK had a negative relationship with that of YL28-L (Figure 2c). In contrast, there was a positive relationship between CK and YL28-L on the PC3 level. The analysis results of β-diversity based on PCoA or nMDS (Supplemental Figure S1) were similar to that by the PCA.

### 3.3. Effects of YL28 Addition on the Bacterial Community Compositions

**Figure 3:**
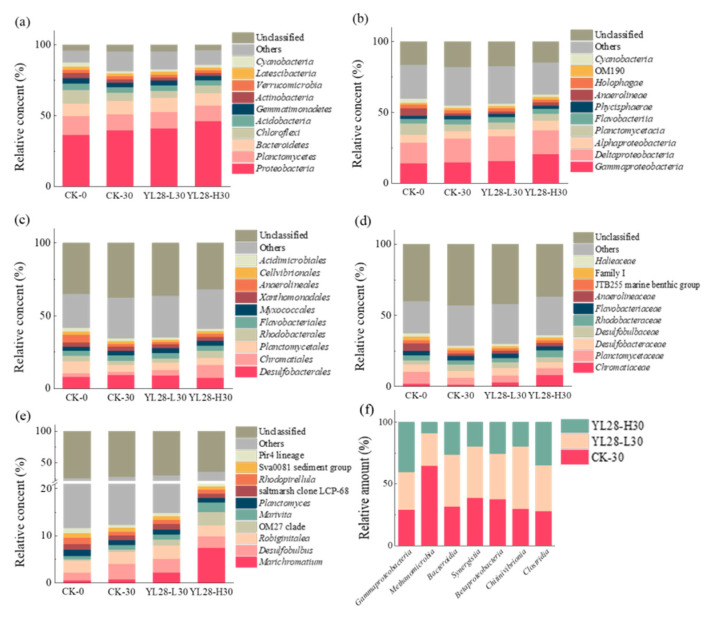
The distribution and composition of bacterial community each classification level. (**a**) Predominant species to relative average abundance statistics at phylum level; (**b**) predominant species to relative average abundance statistics at class level; (**c**) predominant species to relative average abundance statistics at order level; (**d**) predominant species to relative average abundance statistics at family level; (**e**) predominant species to relative average abundance statistics at genus level. Significant difference: *p <* 0.05; (**f**) the relative average abundance changes of significant difference species (*p <* 0.05) at class level.

**Figure 4:**
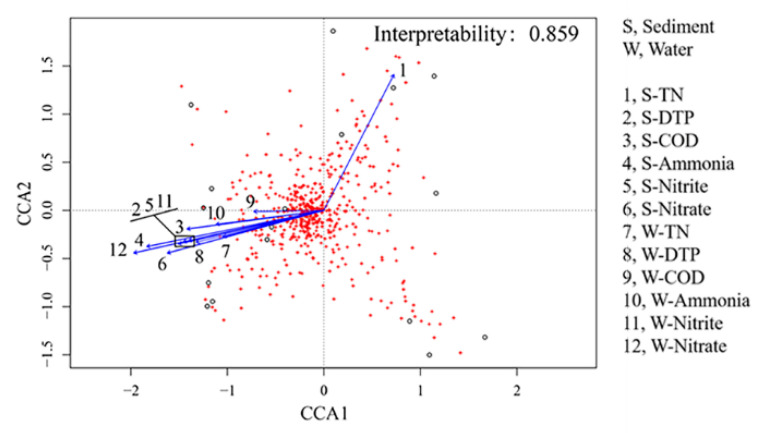
The relationships between environmental factors and bacterial community structure (genus level). Environmental factors include six physicochemical indexes (ammonia, nitrite, nitrate, total nitrogen (TN), dissolved total phosphate (DTP) and chemical oxygen demand (COD)); each was determined in sediments (S) and water column (W); “S” and “W” are used as prefixes to distinguish these indicators.

At the phylum level (Figure 3a, Supplementary Table S2), Proteobacteria was most predominant in all groups (36.53–46.35%), followed by Planctomycete (10.90–13.29%), Bacteroidetes (8.68–10.01%), Chloroflexi (5.11–9.67%), Acidobacteria (3.50–4.73%), Gemmatimonadetes (3.08–3.62%), Actinobacteria (1.99–3.69%), Verrucomicrobia (1.94–2.23%), Latescibacteria (1.73–2.16%) and Cyanobacteria (1.76–2.99%). The relative abundance of other bacterial members occupied approximately 10% of the total populations. The addition of YL28 did not change the predominant bacterial taxa at the phyla level (*p >* 0.05) (Figure 4). However, the relative abundance of Proteobacteria increased at least 16.29% (*p <* 0.05) while the relative abundance of Chloroflexi, Actinobacteria and Latescibacteria decreased less than 10%, compared to the control (*p <* 0.05).

At the class level, Gammaproteobacteria, Deltaproteobacteria and Alphaproteobacteria (Figure 3b, Supplementary Table S3) were the most abundant bacterial members in YL28-H30, which accounted for 20.70%, 16.89% and 6.56%, respectively. There was no significant difference in the abundance of Planctomycetacia and Alpharoteobacteria between CK-30 and YL28-L30 (*p >* 0.05). The relative abundance of the other bacterial members occupied more than 22%. The addition of a higher concentration of YL28 (YL28-H) significantly increased the abundance of Gammaproteobacteria (at least 37.6%) (*p <* 0.05). YL28-H had more Gammaproteobacteria compared to YL28-L, which accounted for over 20% of the total abundance of all experimental groups. 

At the order level, Desulfobacterales (8.15%) and Planctomycetales (8.14%) were the dominant groups in CK-0, indicating a high abundance occurrence of anaerobes such as sulfate-reducing bacteria (SRB) in shrimp mariculture system (Figure 3c, Supplementary Table S4). The supplementation of YL28-H significantly promoted the abundance of Chromatiales (at least 288.04%) (*p <* 0.05).

At the family level (Figure 3d, Supplementary Table S5), the Planctomycetaceae, Desulfobacteraceae and Anaerolineaceae were predominant in CK-0 sample, which accounted for 8.14%, 5.39% and 5.26%, respectively. Compared to CK-0, the abundance of Planctomycetaceae and Anaerolineaceae decreased at least 43.55% and 65.22% (*p <* 0.05) in CK-30, respectively. The addition of a higher concentration of YL28 (YL28-H) led to the increase of 425.33% and 57.44% in Chromatiaceae and Rhodobacteraceae (*p <* 0.05), respectively.

At the genera level, *Robiginitalea*, *Desulfobulbus*, *Rhodopirellula*, *Planctomyces* and Pir4 lineage (Figure 3e, Supplementary Table S6) are predominant in CK-0 group, which accounted for 2.57%, 1.62%, 1.39%, 1.35% and 1.12% of total bacterial populations, respectively. At the end of the observation, *Robiginitalea*, *Desulfobulbus*, and *Planctomyces* were still predominant in CK-30. The relative abundance of *Desulfobulbus* increased 103.83% in CK-30, while there was no significant change in *Robiginitalea* and *Planctomyces* (*p >* 0.05). The relative abundance of *Rhodopirellula* and Pir4 lineage decreased to 0.78% and 0.55% in CK-30, respectively. The unclassified bacteria accounted for up to 63% among the total bacterial population among all the samples. The relative abundance of OM27 increased by 178.16% and 499.57% after the 30-day addition of YL28, respectively (YL28-L vs. YL28-H, *p <* 0.05). Under the same condition, the relative abundance of *Marivita* increased 6.43% and 92.89% in YL28-L30 and YL28-H30, respectively. However, the relative abundance of *Marivita* did not show the significant difference between YL28-L and CK-30 groups (*p >* 0.05).

Remarkably, the addition of YL-28 significantly decreased *Vibrio* OTUs (Supplementary Figure S2). A low dose of YL28 addition had the better inhibition effect for *Vibrio* abundance (62.66% in YL28-L) than did a higher dose (48.85% in YL28-H). Remarkably, the OTU010637 counts decreased 85.35% and 100% in the YL28-L and YL28-H groups, respectively. Moreover, the OTU001940 disappeared in the YL28-H treatment. Analysis of *Vibrio* relative average abundance in different YL28 treatment showed that the inhibition rates of YL28-L and YL28-H on *Vibrio* were 36.36% and 18.18%, respectively.

### 3.4. The Relationships among Physicochemical Parameters, Samples and Bacterial Community

CCA1 and CCA2 explained 85.9% of the total variation (Figure 4). Except for the TN concentration in sediments (S-TN), other eleven environmental factors had the positive relationships to each other. In sediments, the bacterial compositions were mainly influenced by TN, ammonia and nitrate concentrations, which formed two clades as shown in Figure 4. Most of the environmental factors (except TN) had some positive relationships with *Desulfocarbo*, *Desulfatiglans*, *Litoricola*, *Pleurocapsa, Bucillus, Saccharicrinis* and some uncharacterized archaeas taxa (Supplementary Figure S3). Among them, *Desulfocarbo* and *Pleurocapsa* were significantly affected (*p <* 0.05). Moreover, these environmental factors were negatively related to bacteria *Aestuariibacter*, *Maritalea*, *Marivita*, *Marichromatium*, *Parasphingopyxis*, *Polycyclovorans* and *Pseudofulvibacter,* as shown in Supplementary Figure S3. Among them, *Aestuariibacter*, *Marivita*, *Marichromatium* was significantly affected (*p <* 0.05). In water column, the bacterial community structure was only affected by ammonia and nitrate concentrations rather than TN concentration.

### 3.5. The Effects of YL28 on the Abundance of AOB, AOA and the Functional Genes Involved in Nitrogen Metabolisms

**Figure 5:**
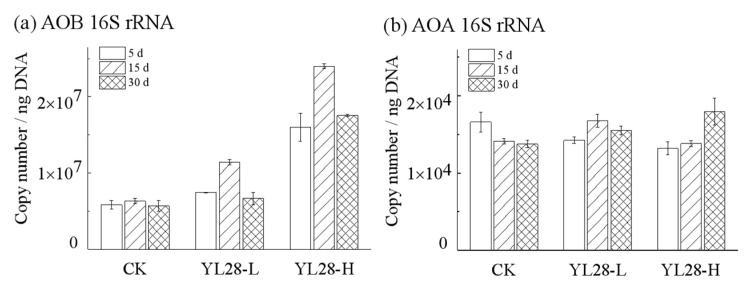
16S rRNA copy number variation in unit mass DNA (ng). (**a**) Copy number per nanogram DNA of ammonia-oxidizing bacteria (AOB). (**b**) Copy number per nanogram DNA of ammonia-oxidizing archaea (AOA). Initial 16S rRNA copy number of AOB and AOA: (5.13 ± 0.46) × 106, 11 073 ± 321. Significant difference: *p <* 0.05.

Compared with the CK groups, the supplementation of YL28 significantly increased the relative abundance of AOB (ammonia-oxidizing bacteria) in YL28-L and YL28-H groups (Figure 5a, *p <* 0.05), but AOA (ammonia-oxidizing archaea) abundance had no significantly change (Figure 5b, *p >* 0.05). On day 5, the abundance of AOB increased by 27.84% in YL28-L and 74.91% in YL28-H treatments when compared to CK (*p <* 0.05). On day 15, the relative abundance of AOB increased by at least 80.34% in YL28-L and 279.75% in YL28-H treatments when compared to CK (*p <* 0.05), respectively. Compared to day 15, the abundance of AOB was reduced significantly at day 30 in YL28-L, which was not significantly different from the CK group (*p >* 0.05).

**Figure 6:**
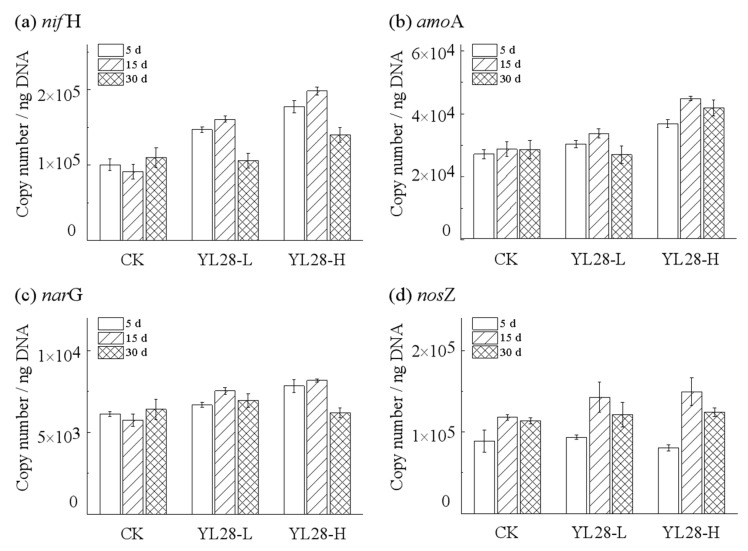
The relative abundance of functional genes involves in nitrification and denitrification during solid waste decomposition. *nif*H: nitrogenase gene (**a**); *amo*A: ammonia monooxygenase gene (**b**); *nar*G: nitrate reductase gene (**c**); *nos*Z: nitrous-oxide reductase Z gene (**d**). These functional genes were predicted by PICRUSt.

Among the tested four functional genes (*nif*H, *amo*A, *nar*G and *nos*Z) involved in nitrogen metabolism, the changes of *nif*H copy numbers were significantly affected by the addition of YL28 (Figure 6a), while the abundance of four genes did not change significantly with time (*p >* 0.05) in CK group. On day 5, the copy numbers of *nif*H increased by 46.32% in YL28-L and 76.84% in YL28-H compared to CK (*p <* 0.05). On day 15, the copy numbers of *nif*H further increased by 76.16% in YL28-L and 117.05% in YL28-H compared to CK (*p <* 0.05). However, the copy numbers of *nif*H were significantly reduced on day 30 compared to day 15, the copy numbers in YL28-H and CK groups were significantly different (*p <* 0.05). The change of *amoA* was similar to that of *nif*H (Figure 6b). The copy numbers of *nar*G and *nos*Z did not change significantly with time (*p <* 0.05) (Figure 6c,d).

### 3.6. Microbiota Function Prediction

**Figure 7:**
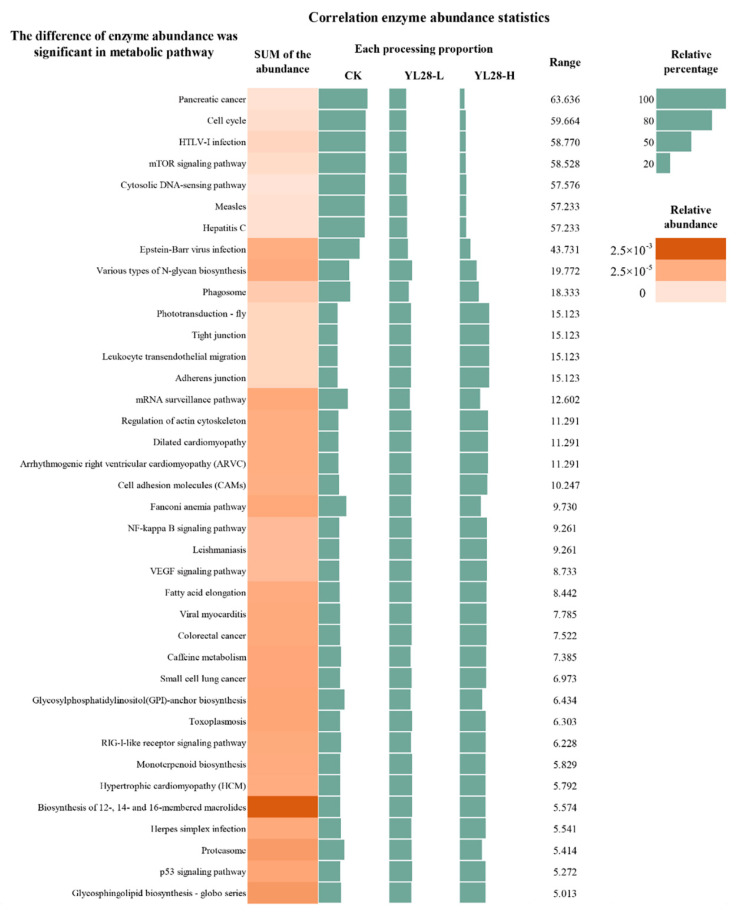
Variation of the average abundance of related enzymes in metabolic pathways (Range > 5%).

To further reveal the relationships between YL28 and bacterial metabolism and circulation in sediment–water interface, microbiota function prediction analysis was conducted based on the method of Tax4Fun. The supplementations of YL28 significantly decreased the enzyme levels related to pancreatic cancer, HTLV-I infection, measles, hepatitis C, phagosome and Epstein–Barr virus infection, which are a serious threat to human health and aquatic animal health (Figure 7, *p <* 0.05). The biggest abundance differences between CK and YL28-H were reached at 63.64% and their decrease was positively correlated with the addition of YL28. The average abundance of enzymes related to environmental information processing and metabolisms accounted for more than 76.01% of the total abundance of enzymes in all samples (Supplementary Figure S4). These pathways were primarily involved in the carbohydrate, amino acid, energy, cofactors and vitamins, nucleotide, lipid and information processing about translation, membrane transport, signal transduction, replication and repair activities. The abundance of enzymes related to nitrogen metabolism accounted for 11%. From the functional analysis, on day 30, a total of 284 groups and 6511 KEGG direct homologous proteins were obtained. The direct homologous proteins of 49.56% had been clearly classified. Redox enzymes, transferase, hydrolase, cleavage enzyme, isomerase and connectors accounted for 12.69%, 15.57%, 11.86%, 4.41%, 2.43% and 2.61% of the total number of homologous proteins, respectively. In the shrimp cultural systems, the abundances of two-component system, ABC transporters, purine metabolism, aminoacyl-tRNA biosynthesis, porphyrin and chlorophyll metabolism, pyrimidine metabolism, nitrogen metabolism, oxidative phosphorylation, ribosome and bacterial secretion system were at least 1.87% (Supplementary Figure S4). The supplementations of YL28 did not change the dominance of these approaches at the level 3. However, in terms of abundance, only methane metabolism (reduced by 6.16%), glycine, serine and threonine metabolism (increased by 4.51%) in the top 30 metabolic pathways showed obvious difference after adding YL28-H (*p <* 0.05).

Analysis of metabolic pathway screened the metabolic pathway-related enzyme abundance groups, which was more than 5% as the research object (Figure 7). The results showed that macrolide (12th, 14th and 16th) had a higher concentration of biosynthesis-related enzymes, reaching 0.245%, followed by sugar phospholipid biosynthesis enzymes (0.063%) and proteases (0.056%). The biosynthesis of sugar-based phosphatidylcholine (GPI) and the p53 signaling pathways occupied 0.0245%. Although the changes of these metabolic pathways were associated with YL28 dosage, it was slightly changed, and the extreme difference was less than 6.97% within groups. The enzyme abundance of other metabolic pathways was less than 0.02% (Figure 7).

## 4. Discussion

### 4.1. Contaminants Removed by YL28 in Aquatic Ecosystems

Excessive generation of pollutants in the depositional environments (such as ammonia, nitrite and phosphate derived from soluble excretions and organic decomposition) leads to eutrophication [22]. It leads to the anoxic conditions which are harmful to benthic biota owing to the oxygen-consuming decomposition [23]. YL28, an excellent probiotic, decreased the ammonia level below 0.01 mg/L, which reached Class I grade Standard of Fisheries and Seawater Water Quality in China (GB3838-2002). YL28 could eliminate or transform toxic nitrogen pollutants which benefited from its 6 nitrogen and 3 sulfur cycle pathways [16]. Due to the significant decrease of ammonia and nitrite level, the growth of pathogens (such as *Vibrio*) and other eutrophic organisms was efficiently inhibited. The relative abundances of the indigenous beneficial microbiota (i.e., *Marichromatium*, *Marivita* and OM27) were significantly increased and *Marichromatium* members could persist in shrimp culture ecosystem for a longer period (up to 6 days). Besides, acute toxicity tests and genetics of ICR mice, the oral toxicity tests of SD mice (28 d) by authority agency in China [13], and bio-toxicity test of *Oryzias melastigma* (marine fish, nearly 300 times higher than normal dose of YL28, data unpublished) showed that YL28 was non-toxic to animals. These results showed that YL28 was a better probiotic which could be safely used in aquaculture.

### 4.2. The Microbial Community Structure in Response to YL28

The microbial community structure was significantly affected by the supplemented probiotics in aquaculture water [24,25,26]. Our results indicated that the supplementation of YL28 significantly changed the bacterial diversity and abundance (Table 1). Even the low-dose addition of YL28 increased the species richness (Table 1). In the PCA and bacterial community structure analysis, a similar bacterial community structure was observed between CK and YL28-L (*p >* 0.05, Table 3). This showed that the lower dosage of YL28 had a smaller effect on bacterial community diversity. Rather, it improved beneficial microbial population (Figure 3f). Compared to CK and YL28-L, YL28-H showed a significant difference (*p <* 0.05) in microbial communities; it was significantly affected by PC1and PC2 (*p <* 0.05). The PCoA analysis further indicated that there was a distinct difference in a high dose of YL28 was applied. YL28-H distinctly clustered separately from these principal coordinates (Supplementary Figure S1).

Proportions of phyla Planctomycetes and Cyanobacteria were different from previous reports [9,27,28,29], highlighting that the different habitat properties of aquaculture ecosystem. The supplementation of YL28 at a high dosage significantly increased the abundance of Proteobacteria, which led to an increase in the population of nitrate- and nitrite-reducing bacteria. It may contribute to the higher nitrogen removal level [27]. On the class level, Zhang et al. showed that the relative abundance of Alphaproteobacteria and Betaproteobacteria increased while Gammaproteobacteria and Deltaproteobacteria decreased when *Rhodopseudamonas palustris* (APB) was added into grass carp culture system [7]. Our results showed that the Gammaproteobacteria abundance significantly increased with the addition of YL28; under the same conditions, the abundance of Alphaproteobacteria and Deltaproteobacteria also slightly increased. It showed that the application of different APB species in the different aquatic culture could lead to microbial structure changes. Duc et al. reported that Planctomycetes and Proteobacteria (class) were dominant groups when marine anammox bacteria were supplemented into the shrimp-mariculture sediment. Compared to the low relative abundance in the original sediment (8%), the abundance of Planctomycetes increased after the additions [29]. Our results also showed that Planctomycetes and Proteobacteria (class) were predominant bacteria groups in all treatments. At the genus level, it is worth noting that the relative abundance of *Marichromatium* genus significantly increased when YL28 was added at a high dose, which was different from a previous report [9]. These results implied that different APB probiotic species had different effects on indigenous bacterial population of ecosystem. It is interesting that the abundances of both *Marivita* and OM27 significantly increased with the addition of YL28. *Marivita* and OM27 were involved in oil degradation in water [30].

### 4.3. The Inhibitory Effect on Growth of Possible Pathogens

Our results showed the relative abundances of pathogen *Vibrio* (62.66% of inhibition rate) significantly decreased when YL28 was supplemented, suggesting that YL28 possibly inhibited the pathogens’ growth in mariculture ecosystem. The intensive shrimp culture often leads to the waste accumulations including residual solid feed, shrimp feces, and soluble excretions, which creates the eutrophic condition. Consequently, the anoxic water favors the fast growth of pathogenic bacteria (such *Vibrio*), which frequently causes the disease outbreaks. The probiotics were proved to inhibit the pathogenic *Vibrio*. For example, *Bacillus subtilis* and *Lactobacillus* spp. inhibited the growth of *Vibrio nereis*, *Vibrio harveyi*, *Vibrio parahaemolyticus* and *Vibrio natriegen* [31,32,33].

### 4.4. The Influence of YL28 on Nitrogen Metabolism-Related Genes

Nitrification and nitrate or nitrite reductions are the central nitrogen cycle pathways during wastewater nitrogen removal. The key enzymes involved in above progresses are the ammonia monooxygenases (encoded by *amo*) [34], the nitrate reductases (encoded by gene *nar*) [35] and nitrous oxide reductases (encoded by gene *nos*Z) [36]. However, it is worth noting that the oceans currently lose substantially more nitrogen by the denitrification; they gain less nitrogen by the transportation from land and in situ N_2_-fixation. Therefore, control of the N loss and the N retention has important implications for the nutrient balance in these ecosystems. The key enzyme involved in N_2_ fixation was nitrogenase encoded by the gene *nif*. The abundances of *amo*A and *nif*H genes were the important signs for maintaining the stability of ecosystem [37]. Among the four tested genes (*nif*H, *amo*A, *nar*G and *nos*Z) involved in nitrogen metabolism, the copy numbers of *nif*H and *amo*A were significantly increased with the addition of YL28 (Figure 6a,b). Copy numbers of *nar*G and *nos*Z had no significant changes with time (Figure 6c,d), but the removal rate of nitrite and nitrate increased (Figure 1b,c), suggesting that the addition of YL28 might stimulate the gene expression involved in assimilation nitrate reduction. This possibly contributes to maintain the nitrogen balance in aquaculture ecosystem [13]. The elevated capacity of the nitrate removal and higher *nos*Z genes abundance were also observed in RuA2-supplemented water samples [9]. Our study demonstrated that the addition of YL28 could significantly improve the nitrogen removal and maintain nitrogen balance of mariculture ecosystem by possibly regulating the gene expression level in nitrogen metabolism.

### 4.5. Altered Metabolic Category in Mariculture Ecosystem by YL28

In this study, the Tax4Fun method was used to analyze the change of microbial metabolism in the sediments of shrimp aquaculture. The result indicated that the addition of YL28 altered the abundance of the metabolic related enzymes in mariculture ecosystem, and increased the abundance of nitrogen metabolism, environmental information processing, the vital metabolism for the bacterial survival and some metabolic pathways related to the growth and health of aquatic animals in ecosystem. Although no link has been established between the health of farmed animals and microbial diversity, some studies have shown that diversity of microflora contributed to animal health and decrease of disease outbreaks [38]. We found that enzymes associated with disease were more enriched in polluted mariculture system (CK group). Whereas, supplementation of YL28 reduced significantly the abundance of enzymes related to diseases (i.e., pancreatic cancer and HTLV-I infection, etc.) and increased significantly the enzyme level related to the growth-promoting for animals (i.e., leukocyte endothelial migration and actin cytoskeleton regulation, etc.). It was speculated that YL28 had a positive preventive effect on the animal diseases. The structure of bacterial community was quite dynamic in different habitats, but there was no significant difference in bacterial metabolism types and the related enzyme diversity. The observation here was consistent with previous studies [38].

## 5. Conclusions

YL28 is a promising probiotic and can be applied safely in aquaculture ecosystem. It could not only efficiently remove inorganic nitrogen (ammonia, nitrite and nitrate) but also stimulated the relative abundances of AOB (ammonia-oxidizing bacteria) and nitrate-and nitrite-reducing bacteria to further remove inorganic nitrogen. Moreover, YL28 significantly reduced the abundance of pathogenic bacteria (*Vibrio*) and the enzyme levels related to diseases. Tax4Fun predictive analysis shows that YL28 could accelerate the process of carbohydrate metabolism, amino acid metabolism, energy metabolism, as well as the growth regulation and disease metabolism of animals, such as actin cytoskeleton regulation. A combined usage of anoxygenic phototrophic bacteria, *Bacillus*, nitrifiers and denitrifiers and *Bdellovibrio* would be a better strategy for different aquaculture ecosystems.

## Figures and Tables

**Table 1:** Richness and diversity indices estimation.

Indices	Sequences per Sample	Control Group	YL28-L	YL28-H
Chao1	49,200	7841.95 ± 896.25	8118.95 ± 350.13	7801.23 ± 895.85
Shannon	49,600	10.64 ± 0.38	10.38 ± 0.38	10.32 ± 0.40

**Table 2:** Significance analysis of Chao1 and Shannon between treatment groups.

Pairwise Comparison	Chao1		Shannon	
	*F* Value	*p* Value	*F* Value	*p* Value
Control group vs. YL28-L	0.247	0.645	0.736	0.439
Control group vs. YL28-H	0.009	0.958	1.074	0.359
YL28-L vs. YL28-H	0.326	0.599	0.039	0.853

**Table 3:** Significance analysis between treatment groups based on Adonis (Permanova) test at genus level.

Pairwise Comparison	Sum of Squares	Mean Square	*F* Value	*R* ^2^	*p*-Value
Control group vs. YL28-L	0.049	0.049	1.939	0.244	0.201
Control group vs. YL28-H	0.405	0.405	15.132	0.716	0.033
YL28-L vs. YL28-H	0.169	0.169	6.281	0.511	0.029

## Data Availability

All data generated or analyzed during this study are available from the corresponding author on reasonable request.

## Supplementary Materials

The following are available online at https://www.mdpi.com/2073-4425/12/1/40/s1. Figure S1: β-diversity analysis of different samples, Figure S2: The inhibition of YL28 to the OUTs number of *Vibrio* (30 d), Figure S3: Heatmap analysis of correlation between environmental factors and species at genus level, Figure S4: Relative average abundance statistics of enzymes in major metabolic pathways of bacteria, Figure S5: Time variation analysis of significant difference species relative abundance at genus level (*P* < 0.05), Table S1: Primer pairs used in amplification of 16S rRNA and functional genes, Table S2: The distribution and composition of bacterial community at the phyla level, Table S3: The distribution and composition of bacterial community at the class level, Table S4: The distribution and composition of bacterial community at the order level, Table S5: The distribution and composition of bacterial community at the family level, Table S6: The distribution and composition of bacterial community at the genera level.
