# Supplementary material for: Effects of Supplement of Marichromatium gracile YL28 on Water Quality and Microbial Structures in Shrimp Mariculture Ecosystems
Source: Genes (Basel). 2020 Dec 30;12(1):40. doi: 10.3390/genes12010040 (PMC7823961; doi:10.3390/genes12010040)
Supplement: Supplementary file 1 [file genes-12-00040-s001.pdf]

Supplemental Materials：

Effects of supplement of *Marichromatium gracile* YL28 on water quality and microbial structures in shrimp mariculture ecosystems

Liang Cui[a, 1], Bitong Zhu[a, 1], Xiaobo Zhang[a], Zhuhua Chan[b], Chungui Zhao[a], Runying Zeng[b], Suping Yang[a, *] Shicheng Chen[c, *],

[a]*Department of Bioengineering and Biotechnology, Huaqiao University, Xiamen, Fujian 361021, China*

[b]*State Key Laboratory Breeding Base of Marine Genetic Resource, Third Institute of Oceanography, ministry of natural resources, No. 178 Daxue Road, Xiamen 361005, China*

[b]*Department of Biomedical Diagnostic and Therapeutic Sciences, School of Health Sciences, Oakland University, Rochester, Michigan. 48309, USA*

Supplemental Materials - Table

Table S1. Primer pairs used in amplification of 16 sRNA and functional genes

Genes	Metabolic Pathways	Primers	Primer Sequences (5'-3')
AOA 16S rRNA	/	16S rRNA-F	GTGYCAGAMGCCGCGGTA
		16S rRNA-R	GGACTACHVGGGTWTCTAAT
AOB 16S rRNA	/	16S rRNA-F	ACGGGGYGCAGCAGGCGCGA
		16S rRNA-R	GGACTACVSGGGTATCTAAT
AOB *nif*H	N_2-NH_4^+	*nif*H-F	AAAGGYGGWATCGGYAARTCCACCAC

. * Corresponding author at: Department of Bioengineering and Biotechnology, Huaqiao University, Xiamen 361021, China.
E-mail address: yangsuping@hqu.edu.cn (Dr. Suping Yang)
. * Corresponding author at: Department of Biomedical Diagnostic and Therapeutic Sciences, School of Health Sciences, Oakland University, Rochester, Michigan. 48309, USA
E-mail address: schen5@oakland.edu (Dr. Shicheng Chen)
[1] These authors contributed to the work equally and should be regarded as co-first authors.

		*nif*H-R	TTGTTSGCSGCRTACATSGCCATCA T
AOB *amo*A	NH_4^+-$NH_3{\cdot}OH^-$	*amo*A-F	GGGGTTTCTACTGGTGGT
		*amo*A-R	CCCCTCKGSAAAGCCTTCTTC
AOB *nos*Z	NO_3^--NO_2^-	*nos*Z-F	CGYTGTTCMTCGACAGCCAG
		*nos*Z-R	CGSACCTTSTTGCCSTYGCG
AOB *nar*G	N_2O-N_2	*nar*G-F	TAYGTSGGGCAGGARAAACTG
		*nar*G-R	CGTAGAAGAAGCTGGTGCTGTT

Table S2. The distribution and composition of bacterial community at the phyla level

Phylum	CK-0	CK-30	YL28-L30	YL28-H30
Proteobacteria	36.530	39.860	41.034	46.352
Planctomycetes	13.286	11.140	11.551	10.903
Bacteroidetes	8.682	9.605	10.011	8.809
Chloroflexi	9.668	5.693	5.110	5.456
Acidobacteria	4.728	3.833	3.728	3.502
Gemmatimonadetes	3.624	3.334	3.490	3.084
Actinobacteria	3.694	2.222	1.987	2.177
Verrucomicrobia	2.232	2.149	2.007	1.940
Latescibacteria	2.161	1.928	2.148	1.728
Cyanobacteria	2.990	1.853	1.764	2.024
Other	8.564	13.882	12.503	10.390
Unclassified	3.843	4.499	4.667	3.636

Table S3. The distribution and composition of bacterial community at the class level

Class	CK-0	CK-30	YL28-L30	YL28-H30
Gammaproteobacteria	14.198	15.040	15.726	20.696
Deltaproteobacteria	14.443	16.729	17.479	16.888
Alphaproteobacteria	5.609	5.177	4.918	6.564
Planctomycetacia	8.157	4.646	4.921	4.832
Flavobacteriia	3.355	3.692	3.704	3.621
Phycisphaerae	2.000	2.447	2.492	2.370
Anaerolineae	5.259	1.829	1.598	2.217
Holophagae	2.497	2.049	2.051	1.886
OM190	1.547	1.729	1.754	1.940
Cyanobacteria	2.737	1.690	1.628	1.887
Other	24.020	27.020	26.443	22.393
Unclassified	16.179	17.953	17.286	14.706

Table S4. The distribution and composition of bacterial community at the order level

Order	CK-0	CK-30	YL28-L30	YL28-H30
Desulfobacterales	8.153	9.307	9.209	7.493
Chromatiales	2.513	2.274	3.633	8.824
Planctomycetales	8.138	4.594	4.914	4.828
Rhodobacterales	3.763	3.073	3.091	4.838
Flavobacteriales	3.355	3.692	3.704	3.621
Myxococcales	3.045	3.205	3.320	3.252
Xanthomonadales	2.730	2.896	2.540	2.807
Anaerolineales	5.259	1.829	1.598	2.217
Cellvibrionales	2.202	1.836	1.724	1.673
Acidimicrobiales	2.628	1.765	1.566	1.668
Other	23.301	28.252	28.587	27.134
Unclassified	34.912	37.275	36.114	31.644

Table S5. The distribution and composition of bacterial community at the family level

Family	CK-0	CK-30	YL28-L30	YL28-H30
Chromatiaceae	2.153	1.583	2.993	8.316
Planctomycetaceae	8.138	4.594	4.914	4.828
Desulfobacteraceae	5.386	4.948	5.102	4.196
Desulfobulbaceae	2.763	4.350	4.098	3.286
Rhodobacteraceae	3.763	3.073	3.091	4.838
Flavobacteriaceae	3.117	3.112	3.318	3.172
Anaerolineaceae	5.259	1.829	1.598	2.217
JTB255 marine benthic group	2.056	2.005	1.818	1.962
Family I	2.706	1.676	1.620	1.878
Halieaceae	2.117	1.731	1.613	1.598
Other	22.595	28.131	28.044	27.094
Unclassified	39.947	42.967	41.790	36.615

Table S6. The distribution and composition of bacterial community at the genera level

Genus	CK-0	CK-30	YL28-L30	YL28-H30
Marichromatium	0.612	0.769	2.217	7.459
Desulfobulbus	1.618	3.298	2.966	2.451
Robiginitalea	2.568	2.521	2.749	2.366
OM27 clade	0.294	0.467	1.299	2.800
Marivita	0.663	1.041	1.108	2.008
Planctomyces	1.353	1.055	1.016	1.039
saltmarsh clone LCP-68	1.155	0.966	1.160	0.874
Rhodopirellula	1.394	0.781	0.860	0.851
Sva0081 sediment group	0.933	0.907	0.875	0.690
Pir4 lineage	1.115	0.550	0.607	0.596
Other	14.493	15.842	15.885	15.175
Unclassified	73.802	71.803	69.259	63.692

Supplemental Materials - Figure

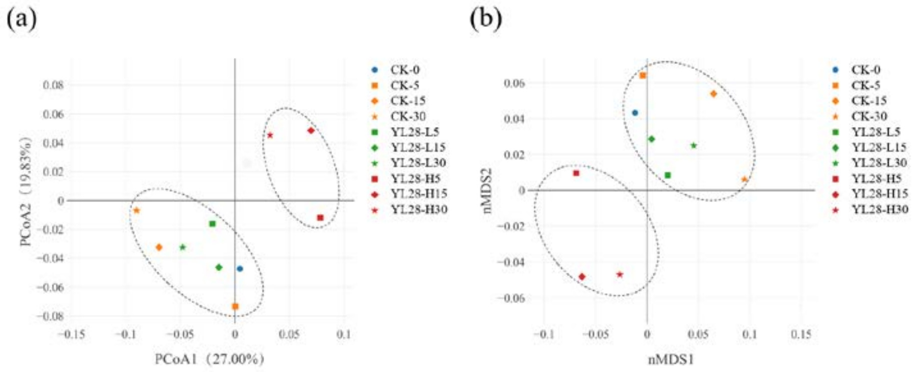

Figure S1. β-diversity analysis of different samples. (a) Principal coordinates analysis (PCoA) of weighted unifrace distance. (b) weighted non-similarity matrix nMDS analysis. Colors and shapes were used to distinguish different systems and samples.

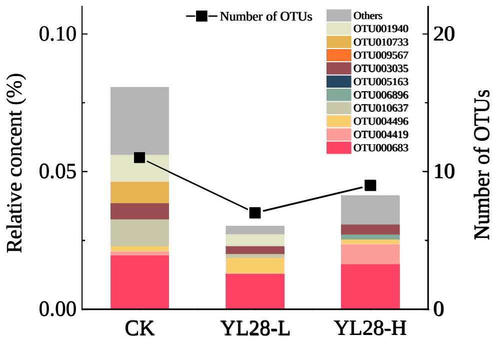

Figure S2. The inhibition of YL28 to the OUTs number of *Vibrio* (30 d). Histogram: dominant OTU sequences of *Vibrio* to relative average abundance statistics in different samples and uses the left Y-axis. Line chart: OTU sequences of *Vibrio* to species statistics and uses the right Y-axis.

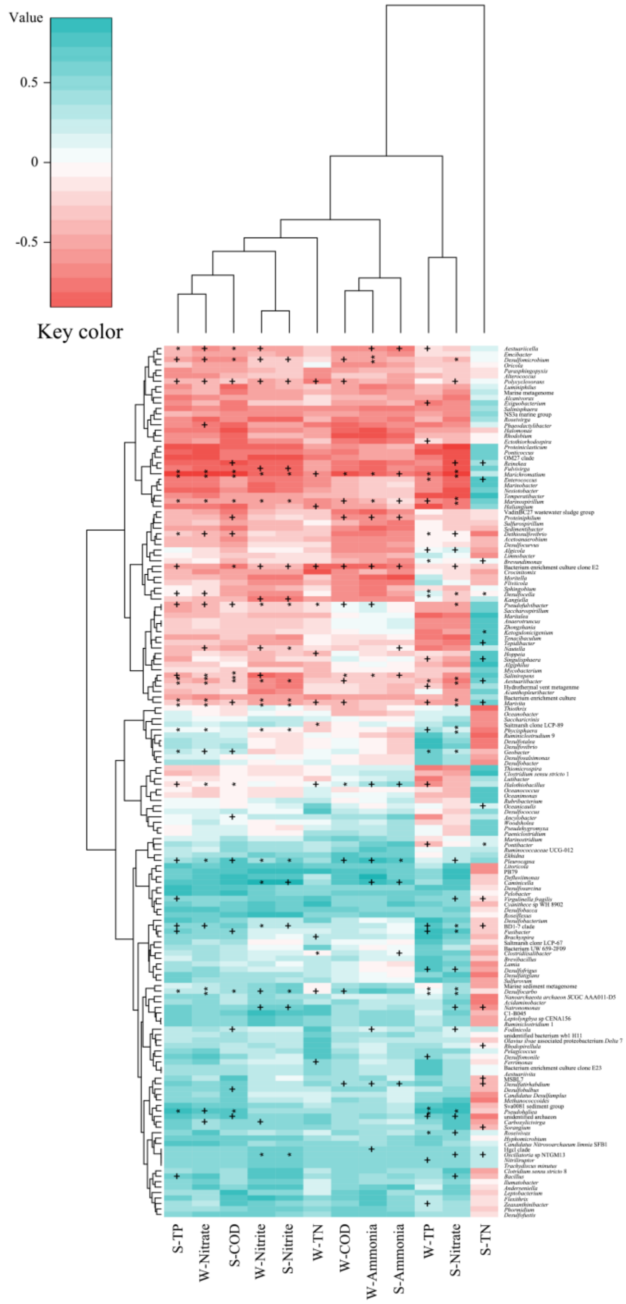

Figure S3. Heatmap analysis of correlation between environmental factors and species at genus level. +, *, ** represent a significant correlation between environmental factors and species abundance at the genus level, respectively. +, $P<0.05$; *, $P<0.01$; ** , $P<0.001$. In the figure, “S” and “W” are used as prefixes to distinguish these indicators, “S” and “W” represent sediment and water column.

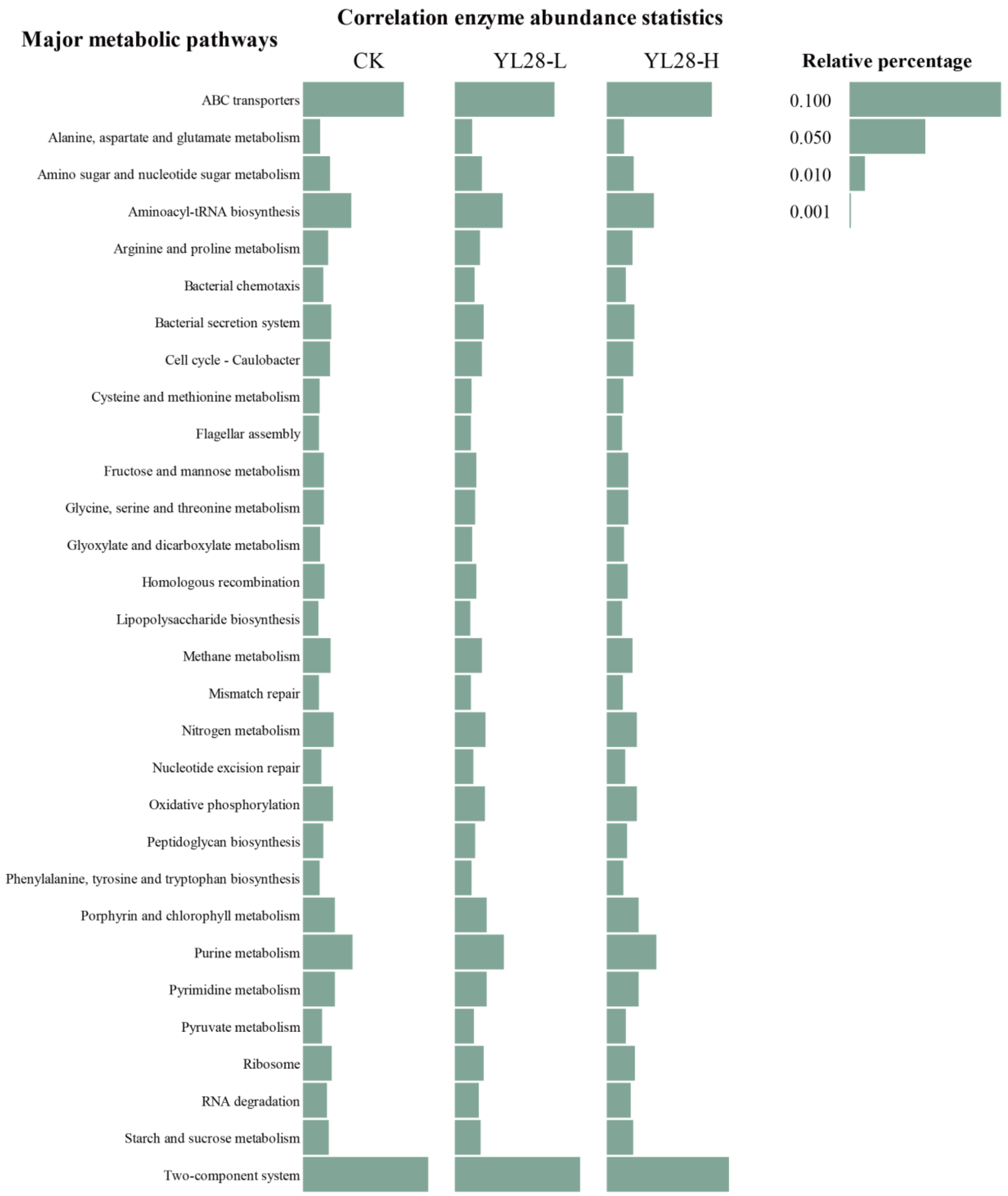

Figure S4. Relative average abundance statistics of enzymes in major metabolic pathways of bacteria

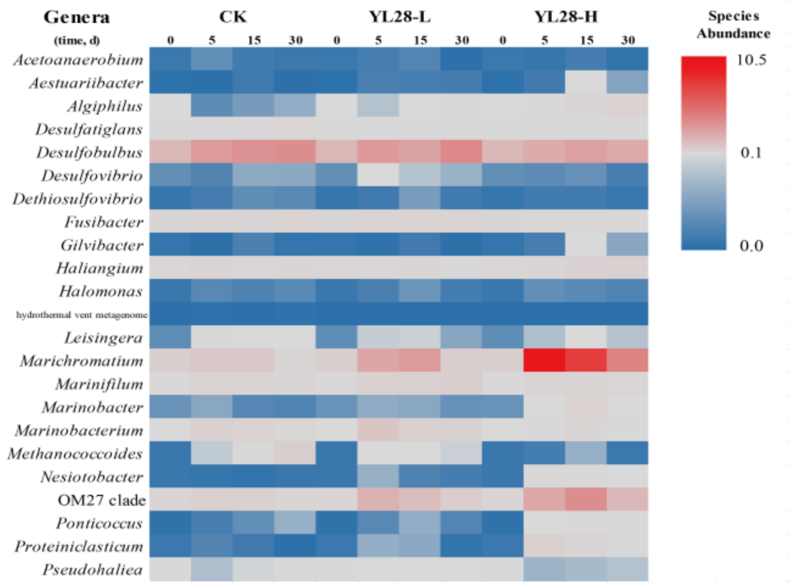

Figure S5. Time variation analysis of significant difference species relative abundance at genus level ($P < 0.05$)
